# Adherence to the DASH Diet and Risk of Hypertension: A Systematic Review and Meta-Analysis

**DOI:** 10.3390/nu15143261

**Published:** 2023-07-24

**Authors:** Xenophon Theodoridis, Michail Chourdakis, Lydia Chrysoula, Violeta Chroni, Ilias Tirodimos, Konstantina Dipla, Eugenia Gkaliagkousi, Areti Triantafyllou

**Affiliations:** 1 Laboratory of Hygiene, Social and Preventive Medicine and Medical Statistics, School of Medicine, Faculty of Health Sciences, Aristotle University of Thessaloniki, 54124 Thessaloniki, Greece; xtheodoridis@auth.gr (X.T.); lchrysoula@auth.gr (L.C.); violetac@auth.gr (V.C.); ityrodim@auth.gr (I.T.); 2 3rd Clinic of Internal Medicine, Papageorgiou Hospital, School of Medicine, Faculty of Health Sciences, Aristotle University of Thessaloniki, 56403 Thessaloniki, Greece; egkaliagkousi@auth.gr (E.G.); artriant@auth.gr (A.T.); 3 Exercise Physiology & Biochemistry Laboratory, Department of Sport Sciences at Serres, Aristotle University of Thessaloniki, 62110 Thessaloniki, Greece; kdipla@phed-sr.auth.gr

**Keywords:** blood pressure, compliance, DASH diet, systematic review, meta-analysis, hypertension

## Abstract

The aim of this study was to assess the effect of the level of adherence to the DASH diet on hypertension risk by conducting a systematic review and meta-analysis. A systematic literature search was performed. Two independent investigators performed the study selection, data abstraction, and assessment of the included studies. The meta-analysis was performed separately with the adjusted hazard (HR) or incident rate ratios (IRR) and the odds ratios (OR) of the highest compared to the lowest DASH diet adherence scores using a random effects model. A total of 12 studies were included in the qualitative and quantitative synthesis. When cohort studies reporting HR were pooled together, high adherence to the DASH diet was associated with a lower risk of hypertension (HR: 0.81, 95% CI 0.73–0.90, *I*^2^ = 69%, PI 0.61–1.08) compared to the low adherence. When cross-sectional studies reporting OR were combined, high adherence to the DASH diet was also related to a lower risk of hypertension (OR: 0.80, 95% CI 0.70–0.91, *I*^2^ = 81%, PI 0.46–1.39). The findings suggest that high adherence to the DASH diet has a positive effect on reducing hypertension risk compared to low adherence. These data strengthen and are in line with all hypertension guidelines, indicating that lifestyle changes should start early even in populations with normal blood pressure.

## 1. Introduction

The prevalence of hypertension doubled in adults older than 30 from 1990 to 2019 [1]. According to the World Health Organization (WHO) [2], it is estimated that almost 50% of adults with hypertension are undiagnosed. Prediction models project that in 2030 approximately 40% of adults in the U.S.A. will develop some form of cardiovascular disease (CVD), including hypertension [3]. To tackle this public health issue, the WHO set a global target to ameliorate the prevalence of hypertension by ¼ by 2025 [4].

High blood pressure is the leading cause of disability-adjusted life years (DALY) [5] and accounts for most of the CVD events worldwide [6] and premature deaths [7]. Even though prevention policies are of fundamental importance to mitigate the tremendous increase in hypertension rates and reduce the development of related comorbidities, most clinical practice guidelines focused primarily on the treatment of this condition [8,9]. The recommendations for preventing hypertension include the amendment of dietary deviations from guidelines and physical inactivity [10]. Decreasing the amount of sodium intake and the achievement of weight loss for adults with overweight or obesity are strategies that will prevent hypertension-attributed deaths and reduce the risk of hypertension, respectively [6].

The most studied dietary pattern for high blood pressure is the Dietary Approaches to Stop Hypertension (DASH) eating plan. The DASH diet has been proposed for the management of high blood pressure due to the inclusion of food groups with antihypertensive properties [11]. More specifically, the DASH diet emphasizes the consumption of fruits, vegetables, whole grains, legumes, nuts, lean protein, and low-fat dairy products. Furthermore, it focuses on limited intakes of salt, added sugar, and saturated fat. Many studies have assessed whether the level of adherence to the DASH diet can reduce hypertension risk among the adult population, with inconclusive results [12,13,14,15].

To the best of our knowledge, there is not a published systematic review and meta-analysis assessing the effect of the level of adherence to the DASH diet on the development of hypertension. Hence, the objective of this work was to synthesize all the data from the available primary studies to shed light on the inconclusive results.

## 2. Materials and Methods

### 2.1. Protocol and Registration

The present systematic review and meta-analysis has been conducted according to the Preferred Reporting Items for Systematic Reviews and Meta-Analyses (PRISMA 2020) [16] and Meta-analysis Of Observational Studies in Epidemiology (MOOSE) [17] statements (Supplementary Tables S1 and S2). A pre-specified protocol has been registered in the Prospero repository (CRD42022344686).

### 2.2. Search Strategy

A comprehensive literature search was conducted in MEDLINE via PubMed, Scopus, and Web of Science Core Collection databases from inception to November 2022 by two independent reviewers. Furthermore, we inspected the references of the included studies for relevant articles. The grey literature was also searched for potential records. Finally, we consulted experts in the field of nutrition and hypertension for the provision of eligible studies. We used search terms such as “hypertension”, “blood pressure”, “DASH diet”, and “dietary adherence”. The full search string can be found in Supplementary Table S3.

### 2.3. Study Selection

We included records that met the following criteria: (1) observational and/or interventional studies, (2) including adult population without a hypertension diagnosis, (3) comparing the effect of high and low adherence to the DASH diet, (4) on the risk of developing hypertension.

Adherence to the DASH diet is defined as the degree to which an individual follows the DASH diet. We defined hypertension according to ESC/ESH guidelines [8], namely, systolic blood pressure (SBP) ≥ 140 or/and diastolic blood pressure (DBP) ≥ 90 or the use of antihypertensive medication.

Studies including pregnant or pediatric populations or those written in a non-English language were excluded.

### 2.4. Data Extraction

We independently abstracted data regarding study characteristics including the first author’s name, publication year, the country in which the study took place, study design, study population details, comorbidities, DASH diet assessment tool, and the use of anti-hypertensive medication. As far as statistical data are concerned, we independently extracted risk estimates with their corresponding 95% confidence intervals (CI) regarding the risk of hypertension based on the level (high or low) of adherence to the DASH diet.

### 2.5. Risk of Bias in Individual Studies

Two independent researchers assessed the risk of bias in the included observational studies using the Newcastle–Ottawa scale (NOS) and checklists for cross-sectional or cohort studies developed by the Joanna Briggs Institute (JBI). The JBI checklist for the cohort studies includes 11 items regarding the study design, while the checklist for the cross-sectional studies comprises 8 questions. There are three available options to respond to these items, “yes” indicating high quality, “no” indicating poor quality, or “unclear”.

### 2.6. Data Synthesis

To answer our research question, we conducted two statistical analyses, one including only cohort studies and another one including only cross-sectional studies. The meta-analysis was performed separately for the adjusted hazard ratios (HR) or incident rate ratios (IRR) and odds ratios (OR) of the highest compared to the lowest DASH diet adherence score using a random effects model. The heterogeneity was estimated using the estimator proposed by Paule and Mandel [18], and measured using the *I*^2^ index, which describes the percentage of variability due to heterogeneity rather than sampling error, and the τ^2^ [19]. We present the prediction interval (PI), which facilitates clinical interpretation of the heterogeneity and quantifies the range of the effect size that a future study will fall [20]. Funnel plots and publication bias tests for assessing their asymmetry were not calculated due to the few included studies [21]. We also performed a subgroup and sensitivity analysis in order to explain heterogeneity and assess the robustness of our findings, respectively. Data were analyzed using the R Studio software (version 2023.06.0) and meta package. Statistical significance was set at *p* < 0.05.

### 2.7. Quality of the Evidence

The certainty of the evidence of our findings was assessed using the Grading of Recommendations, Assessment, Development, and Evaluations (GRADE) approach, as recommended by the Cochrane Handbook [19].

## 3. Results

### 3.1. Database Search and Study Characteristics

**Figure 1:**
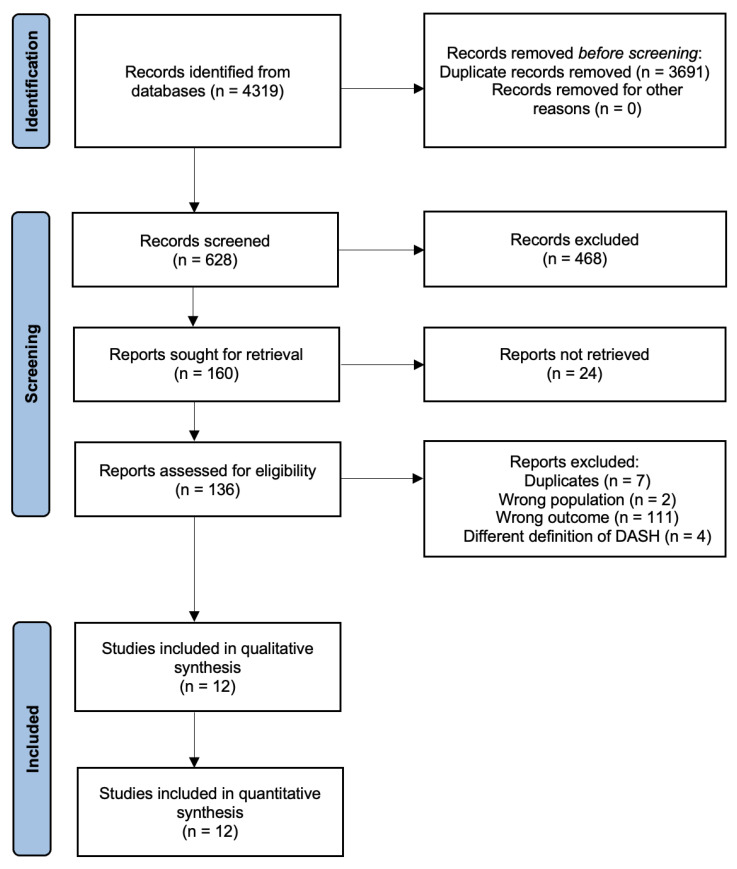
Flow diagram of the eligibility process.

An electronic literature search was performed on MEDLINE via PubMed, Scopus, and Web of Science Core Collection, and a total of 4319 articles were retrieved. After the duplicate removal process, 628 records remained for further evaluation. After title and abstract screening, 136 articles were assessed for eligibility. The final sample of the study incorporated 12 individual studies [14,15,22,23,24,25,26,27,28,29,30,31]. Figure 1 presents the details of the study search and selection process.

The main characteristics of the included studies are summarized in Table 1 and Table 2. Briefly, of the total 12 studies, three were cross-sectional and nine were cohort studies. The total number of participants exceeded 115,000 and their mean age ranged from 36.3 to 61.0 years old.

### 3.2. Definitions of the DASH Diet

Four studies used the definition of the DASH diet based on the DASH score constructed by Fung and colleagues [32], while six studies provided their own definition of the DASH diet score based either on food groups or types of macro- and micronutrients using different cut points for low and high adherence. One study defined the DASH diet according to recommended and restricted food groups as well as sodium consumption based on the guide published by the National Institutes of Health and the National Heart Lung and Blood Institute. In one study, the DASH diet was described as the sum of three food groups, namely, vegetables, fruit, and milk products using the hypothesis-oriented pattern variable.

### 3.3. Outcome of Interest

**Figure 2:**
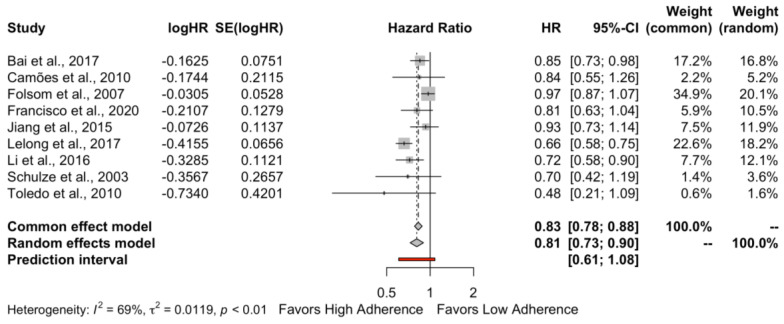
Forest plot for the hypertension risk when cohort studies were pooled together. Bai et al., 2017 [22], Camões et al., 2010 [23], Folsom et al., 2007 [24], Francisco et al., 2020 [25], Jiang et al., 2015 [14], Lelong et al., 2017 [26], Li et al., 2016 [27], Schulze et al., 2003 [28], Toledo et al., 2010 [29].

When the cohort studies reporting HR were pooled together (Figure 2), high adherence to the DASH diet was associated with a lower risk of hypertension (HR: 0.81, 95% CI 0.73–0.90, *I*^2^ = 69%, PI 0.61–1.08) compared to low adherence. Based on the Cochrane Handbook, the heterogeneity appears to be substantial.

**Figure 3:**
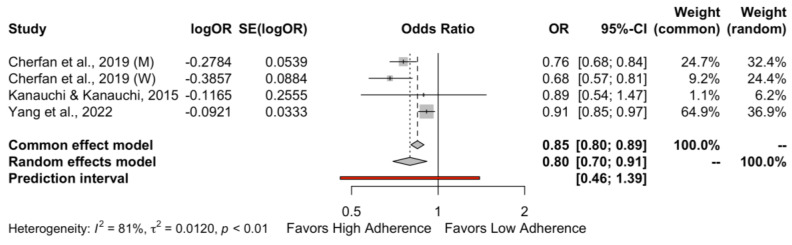
Forest plot for the hypertension risk when cross-sectional studies were pooled together. Cherfan et al., 2019 (M) [30], Cherfan et al., 2019 (W) [30], Kanauchi & Kanauchi, 2015 [15], Yang et al., 2022 [31].

When cross-sectional studies reporting OR were combined (Figure 3), high adherence to the DASH diet was not related to the risk of hypertension (OR: 0.80, 95% CI 0.70–0.91, *I*^2^ = 81%, PI 0.46–1.39). A considerable heterogeneity was observed for the DBP outcome.

### 3.4. Risk of Bias Assessment

The results of the assessment using the NOS are presented in Supplementary Tables S4 and S5. The quality assessment of the cross-sectional and cohort studies based on the JBI checklists are presented in Supplementary Tables S6 and S7, respectively. For the cross-sectional studies, the articles did not provide enough information about the study subjects and the setting. Regarding the appraisal of the cohort studies, only two studies [22,27] had sufficient follow-up time for the outcome of interest to occur.

### 3.5. Subgroup and Sensitivity Analysis

We conducted a subgroup analysis based on the hypertension diagnosis (i.e., SBP ≥ 140 and/or DBP ≥ 90 or antihypertensive medication use versus self-report of hypertension). The results of the subgroup analysis indicate that there is no difference between the two methods used for hypertension diagnosis. Specifically, the pooled estimate for diagnosis of hypertension based on values of SBP ≥ 140 and/or DBP ≥ 90 or the use of antihypertensive medication was HR: 0.85 (95% CI 0.77–0.95, *I*^2^ = 0%). On the other hand, the summary effect for the self-report of hypertension method was HR: 0.75 (95% CI 0.60–0.95, *I*^2^ = 88%) (Supplementary Figure S1).

A sensitivity analysis has also been conducted to assess the robustness of the findings. In this analysis, cohort studies with a NOS score < 7 were removed. The results of the sensitivity analysis were HR: 0.81, 95% CI 0.71–0.92, *I*^2^ = 4%, PI 0.64–1.01 (Supplementary Figure S2).

### 3.6. Certainty of the Evidence

According to the GRADE approach, the quality of our evidence was deemed very low for both effect sizes (i.e., HR and OR). Risk of bias, inconsistency, indirectness, and imprecision were the domains that both comparisons were downgraded by one level.

## 4. Discussion

The present systematic review and meta-analysis aimed to evaluate the effect of the level of adherence to the DASH diet on hypertension risk. The findings suggest that, based on the pooled estimate from the cohort studies, high adherence to the DASH diet has a positive effect on hypertension prevention compared to low adherence. This observation is in line with the findings resulting from the data of the cross-sectional studies that were also synthesized.

With respect to potential antihypertensive mechanisms of the DASH diet, decreased sodium and increased potassium intake are among the most well-studied factors. Specifically, the DASH diet is rich in fruits and vegetables with high amounts of potassium, which shows vasoactive properties and possibly reduces blood pressure through a decrease in vascular smooth muscle contraction [33]. On the other hand, potassium increases urinary sodium excretion and reduces insulin resistance and oxidative damage [25]. Insulin resistance with compensatory hyperinsulinemia and reactive oxygen species that influence the homeostasis of the vascular wall could lead to hypertension [34,35].

On the contrary, high sodium diets lead to water retention, which, in turn, causes an expansion in circulating volumes, a rise in cardiac output, and an increase in kidney perfusion pressure [36]. Moreover, high kidney perfusion pressure prompts a rise in the glomerular filtration rate and sodium excretion in order to restore body fluids. Another plausible mechanism is that excessive sodium intake elicits a reduction in vascular nitric oxide concentration, which is responsible for endothelium-dependent dilation [37].

High dietary sodium intake is associated with arterial stiffness mainly due to a modification in the extracellular matrix of the arterial wall [38,39]. A J-shaped curve has been found to resemble the relationship between sodium or potassium intake and vascular structure and function [40]. Evidence supports that arterial stiffness is related to a higher risk of hypertension incidence [41].

An increase in dietary fiber intake has also been associated with a reduction in both systolic and diastolic blood pressure [42]. The reduction in blood pressure depends on the type of dietary fiber, where β-glucan appears to be the most effective one [43]. An improvement of insulin sensitivity and endothelial function, stimulation of the absorption of minerals in the gastrointestinal tract, and reduction in body weight are among the mechanisms that have been proposed to link fiber intake and blood pressure control [44].

A systematic review and meta-analysis of randomized controlled trials demonstrated that the DASH diet reduces blood pressure in both normotensive and hypertensive adults [11]. This study also showed that the blood pressure-lowering effect of the DASH diet was more prominent in participants aged <50 years and among those with a sodium intake >2400 mg/d [11]. Another recently published systematic review and meta-analysis of randomized controlled trials found that a modified DASH diet is effective in decreasing blood pressure and some cardiometabolic markers, such as waist circumference and triglyceride concentration in patients with hypertension [45]. From this study, a higher baseline blood pressure is linked to more pronounced systolic and diastolic blood pressure decreases [45]. Finally, another systematic review and dose-response meta-analysis by Soltani and colleagues [46] indicated that even a low adherence to the DASH diet was associated with lower all-cause, cardiovascular, and cancer mortality.

Our findings showed that high adherence to the DASH diet has a protective role on the risk of hypertension in comparison with low adherence. Even though the pooled estimates from the cohort and cross-sectional studies are in agreement, findings derived from the cross-sectional studies should be interpreted with more caution, as they are at a lower level of the evidence hierarchy compared to the cohort studies [47]. Hence, these studies may have less methodological rigor and more biases affecting their conclusions. This is also supported by the wider PI emerging from the synthesis of the cross-sectional studies when compared to the PI resulting from the pooling of the cohort studies [48].

To further explore the substantial heterogeneity presented in the synthesis of the cohort studies, a subgroup analysis based on the hypertension diagnostic method was performed. The results of this analysis showed that there was no statistical heterogeneity between studies that used the most accurate diagnostic method for hypertension. Contrarily, high heterogeneity was still present in the studies that used self-reporting of hypertension as the method of their choice.

The results of the sensitivity analysis are in line with the results of our primary analysis, indicating that our findings are robust. Furthermore, upon exclusion of the cohort studies deemed of low quality based on the NOS assessment, a reduction in the heterogeneity of the summary effect to an *I*^2^ = 4% was observed. This reduction indicates the absence of heterogeneity among the included studies.

The findings of the present systematic review indicate the beneficial effect of high adherence to the DASH diet on the risk of developing hypertension in subjects with normal blood pressure values. Healthcare professionals including doctors, dietitians, and nurses, as well as policy-makers, should recommend early compliance to the basic guidelines of the DASH diet in order to reduce the incidence of hypertension and the related comorbidities. Future studies should prioritize the development and validation of an instrument assessing adherence to the DASH diet, which could be utilized in research trials. Upon such a successful acceptance from the scientific society, it could then be also applied to the clinical setting. Additionally, larger sample sizes studies encompassing diverse participants are welcomed.

Compliance with the Cochrane guidelines, the rigor of statistical and methodological aspects used, and that this is the first systematic review and meta-analysis assessing the effect of the level of adherence to the DASH diet on hypertension risk in normotensive individuals are some of the strengths of our study. However, there are limitations that should be accounted for. Firstly, the low quality of the included observational studies reduces the certainty of the evidence. Furthermore, some studies reported hazard ratios while others reported odds ratios; hence, we could not pool data from all the available studies. Another limitation is that the included studies defined hypertension and DASH diet adherence based on different thresholds and scores, respectively. Lastly, the inclusion of studies written in the English language can only comprise a limitation of our study. However, two meta-epidemiologic studies showed that restricting evidence synthesis to English-language articles has a modest effect on effect estimates and the study’s conclusion [49,50].

## 5. Conclusions

The findings suggest that high adherence to the DASH diet has a positive effect on reducing hypertension risk compared to low adherence. These data strengthen and are totally in line with all hypertension guidelines, i.e., European, American, and International, independent of the cut-off points used to define hypertension, pointing out that lifestyle modifications should start early before the establishment of hypertension, even in subjects with normal blood pressure levels.

## Figures and Tables

**Table 1:** Characteristics of the included studies.

Study ID, Country	Study Design, Effect Size	Population	No of Participants (Low/High Adherence)	Mean Age ± SD	Exclusion Criteria	DASH Assessment Tool	Hypertension Diagnosis
Bai et al., 2017 [22], China	Longitudinal-cohort, HR	Chinese adults	-	42 ± 9.3	Younger than 18 years old, missing average SBP or DBP, identified hypertension, antihypertensive medication, existing diagnosis of diabetes, myocardial infarction, or apoplexy	DASH diet score Fung et al. (2008) [32]	SBP ≥ 140 or DBP ≥ 90 or antihypertensive medication use
Camões et al., 2010 [23], Portugal	Longitudinal cohort study, HR	Portuguese adults, resident in Porto and at risk of developing hypertension	246/256	-	Age < 39 years old, missing information on BP measurements, hypertensive	Developed DASH diet score	SBP ≥ 140 or DBP ≥ 90 or antihypertensive medication use
Cherfan et al., 2019 [30], France	Cross-sectional analysis, OR	Adult workers or retired	3709/29,342	-	BMI < 18 kg/m^2^	Constructed DASH diet score according to Fung et al. (2008) [32]	SBP ≥ 140 or DBP ≥ 90 or antihypertensive medication use
Folsom et al., 2007 [24], U.S.	Cohort study, HR	Adult women	5017/4041	61.0	History of physician-diagnosed hypertension, heart attack, angina, heart disease, diabetes, more than 29 blank items on FFQ, EI < 500 kcal or >5000 kcal, missing covariates	Developed DASH diet index score	Self-report of hypertension
Francisco et al., 2020 [25], Brazil	Longitudinal cohort study, HR	Adults active or retired civil services of higher research institutions located in Brazil	4987/645	49.9 ± 8.3	Hypertension, antihypertensive drugs, CVD, changed dietary habits in the last 6 months, self-reported their race/skin color as Asian, Brazilian indigenous, missing information on BP values	Dash diet score calculated by National Institutes of Health, National Heart Lung and Blood Institute	SBP ≥ 140 or DBP ≥ 90 or antihypertensive medication use
Jiang et al., 2015 [14], U.S.	Longitudinal cohort study, HR	U.S. adults	585/331	52.5 ± 9.4	Medication, no SBP or DPB recorded at follow-ups, no valid FFQ, missing information for hypertension medication	Developed DASH diet score using score by Folsom et al. (2007) [24]	SBP ≥ 140 or DBP ≥ 90 or antihypertensive medication use
Kanauchi et al., 2015 [15], Japan	Cross-sectional, OR	Male workers	-	45.3 ± 6.9	Diabetes, CKD, implausibly low or high estimated EI, missing information	Developed DASH diet score	SBP ≥ 140 or DBP ≥ 90
Lelong et al., 2017 [26], France	Prospective cohort study, HR	Adults internet user volunteer	19,967/ 19,323	41.9 ± 14.0	Energy under reporters, with < 3 24 h valid recalls, prevalent hypertension, cancer, diabetes mellitus, and cardiovascular disease, pregnant women, missing or invalid data on health status, anthropometric measurements, or physical activity	DASH diet score Fung et al. (2008) [32]	Self-report of hypertension
Li et al., 2016 [27], U.S.	Cohort study, HR	Adult women	706/747	36.5 ± 4.3	History of cardiovascular disease, cancer, multiple gestations or pregnancies lasting <6 months, history of GDM, history of hypertension beforethe diagnosis of GDM or with missing data on post-pregnancy diet	DASH diet score Fung et al. (2008) [32]	Self-report of hypertension
Schulze et al., 2003 [28], Germany	Cohort study, HR	Women in the EPIC-Potsdam Study	-	-	Previous diagnosis of hypertension, antihypertensive medication within a 4-week period prior to the baseline examination, missing information on dietary intake, estimated basal metabolic rate, physical activity, lifestyle characteristics, and anthropometric measurements; current pregnancy or breastfeeding, outlying total energy intake, with no follow-up, possible hypertension for whom we did not have completed verification, prevalent or secondary hypertension	DASH diet score based on hypothesis-oriented pattern variable	-
Toledo et al., 2010 [29], Spain	Prospective cohort study, HR	University graduates	6487/158	36.3 ± 11.0	Self-reported prevalent hypertension with extreme total EI, prevalent CVD at baseline	Developed DASH diet score	Self-report of hypertension
Yang et al., 2022 [31], China	Cross-sectional, OR	Chinese adults	12,298/11,862	-	Incomplete dietary information, incomplete basic information, incomplete physical examination and laboratory test, implausible dietary EI < 500 kcal/d or >5000 kcal/d, and pre-diagnosed coronary heart disease or stroke	Developed DASH diet score	SBP ≥ 140 or DBP ≥ 90 or antihypertensive medication use

BMI: Body Mass Index; BP: Blood Pressure; CKD: Chronic Kidney Disease; CVD: Cardiovascular Disease; DASH: Dietary Approaches to Stop Hypertension; DBP: Diastolic Blood Pressure; EI: Energy Intake; EPIC: European Prospective Investigation into Cancer and Nutrition; ESC/ESH: European Society of Cardiology/European Society of Hypertension; FFQ: Food Frequency Questionnaire; GDM: Gestational Diabetes Mellitus; HR: Hazard Ratio; OR: Odds Ratio; SBP: Systolic Blood Pressure; SD: Standard Deviation.

**Table 2:** Patients’ health characteristics of the included studies.

First Author, Year	BMI	SBP	DBP	Physical Activity	Smoking Status	Sodium Intake	Potassium Intake (Low/High)
	(Low/High)	(Low/High)	(Low/High)	(Low/High)	(Low/High)	(Low/High)	
Bai et al., 2017 [22]	NA	NA	NA	NA	NA	NA	NA
Camões et al., 2010 [23]	NA	NA	NA	NA	NA	NA	NA
Cherfan et al., 2019 [30]	NA	NA	NA	NA	NA	NA	NA
Folsom et al., 2007 [24]	26.3/25.3	NA	NA	16.0%/40.0%	Current smokers = 22.0%/10.0%	2124.0 mg/d 2275.0 mg/d	1147.0 mg/d 1437.0 mg/d
				high PA *			
Francisco et al., 2020 [25]	25.8 ± 4.2/ 24.9 ± 3.8	114.5 ± 11.5/ 114.5 ± 11.8	72.7 ± 8.1/ 71.4 ± 8.2	Low Adherence:	Low Adherence:	4.6 ± 14.4 g/d 3.5 ± 3.0 g/d	3982.0 ± 1607.0 mg/d 5260.0 ± 1664.0 mg/d
				Light = 78.6%	Non-smoker = 58.8%		
				Moderate = 14.1%	Former smoker = 25.8%		
				High = 7.3%	Smoker = 15.4%		
				High Adherence:	High Adherence:		
				Light = 62.8%	Non-smoker = 65.3%		
				Moderate = 24.8%	Former smoker = 25.4%		
				High = 12.4%	Smoker = 9.3%		
Jiang et al., 2015 [14]	27.1/25.9	121.1/119.0	73.6/71.7	35.6/34.6 PAI	35.9%/7.0%	1145.3/1000 kcal	1468.3/1000 kcal
						1146.0/1000 kcal	1902.2/1000 kcal
Kanauchi et al., 2015 [15]	NA	NA	NA	NA	NA	NA	NA
Lelong et al., 2017 [26]	23.8 ± 4.7/ 22.7 ± 3.6	NA	NA	Low Adherence:	Low Adherence:	2907.0 ± 958.0 mg/d 2454.0 ± 857.0 mg/d	2623.0 ± 726.0 mg/d 3409.0 ± 884.0 mg/d
				Low = 31.3%	Never = 48.7%		
				Moderate = 41.5%	Former Smoker = 25.8%		
				High = 27.3%	Current = 25.6%		
				High Adherence:	High Adherence:		
				Low = 17.4%	Never = 53.6%		
				Moderate = 44.1%	Former Smoker = 36.1%		
				High = 38.5%	Current = 38.5%		
Li et al., 2016 [27]	26.8 ± 6.5/ 25.8 ± 5.7	NA	NA	12.5 ± 18.3/21.9 ± 25.4	19.0%/7.0%	NA	NA
				(MET × h/week)			
Schulze et al., 2003 [28]	NA	NA	NA	NA	NA	NA	NA
Toledo et al., 2010 [29]	23.0 ± 3.0/ 23.0 ± 3.0	NA	NA	23.5 ± 20.9/32.1 ± 30.1 (MET × h/week)	Low Adherence:	3.4 ± 2.2 g/d 3.1 ± 1.5 g/d	4.3 ± 1.2 g/d 7.3 ± 2.1 g/d
					Current = 25.0%		
					Ex-smokers = 25.0%		
					High Adherence:		
					Current = 15.0%		
					Ex-smokers = 30.0%		
Yang et al., 2022 [31]	NA	NA	NA	NA	NA	NA	NA

MET: Metabolic Equivalent of Task; NA: Not Available; PA: Physical Activity; PAI: Physical Activity Index. * No definition of high PA.

## Data Availability

The data presented in this study are available in the main text and Supplementary Materials.

## Supplementary Materials

The following supporting information can be downloaded at: https://www.mdpi.com/article/10.3390/nu15143261/s1, Supplementary Table S1. PRISMA 2020 checklist. Supplementary Table S2. MOOSE checklist. Supplementary Table S3. Search strategy for identifying studies on Pubmed. Supplementary Table S4. Quality assessment using the Newcastle-Ottawa scale for cross-sectional studies. Supplementary Table S5. Quality assessment using the Newcastle-Ottawa scale for cohort studies. Supplementary Table S6. Quality assessment using the JBI checklist for cross-sectional studies. Supplementary Table S7. Quality assessment using the JBI checklist for cohort studies. Supplementary Figure S1. Subgroup analysis for the cohort studies based on hypertension diagnosis. Supplementary Figure S2. Sensitivity analysis by removing cohort studies with a NOS score < 7.
