# Supplementary material for: Adherence to the DASH Diet and Risk of Hypertension: A Systematic Review and Meta-Analysis
Source: Nutrients. 2023 Jul 24;15(14):3261. doi: 10.3390/nu15143261 (PMC10383418; doi:10.3390/nu15143261)
Supplement: Supplementary file 1 [file nutrients-15-03261-s001.zip › nutrients-2505372-supplementary.pdf]

Contents

Supplementary Table S1. PRISMA 2020 checklist

Supplementary Table S2. MOOSE checklist

Supplementary Table S3. Search strategy for identifying studies on Pubmed

Supplementary Table S4. Quality assessment using the Newcastle-Ottawa scale for cross-sectional studies

Supplementary Table S5. Quality assessment using the Newcastle-Ottawa scale for cohort studies

Supplementary Table S6. Quality assessment using the JBI checklist for cross-sectional studies

Supplementary Table S7. Quality assessment using the JBI checklist for cohort studies

Supplementary Figure S1. Subgroup analysis for the cohort studies based on hypertension diagnosis

Supplementary Figure S2. Sensitivity analysis by removing cohort studies with a NOS score <7

Supplementary Table S1. PRISMA 2020 checklist

Section and Topic	Item #	Checklist item	Location where item is reported
**TITLE**			
Title	1	Identify the report as a systematic review.	Page 1
**ABSTRACT**			
Abstract	2	See the PRISMA 2020 for Abstracts checklist.	Page 1
**INTRODUCTION**			
Rationale	3	Describe the rationale for the review in the context of existing knowledge.	Pages 1-2
Objectives	4	Provide an explicit statement of the objective(s) or question(s) the review addresses.	Page 2
**METHODS**			
Eligibility criteria	5	Specify the inclusion and exclusion criteria for the review and how studies were grouped for the syntheses.	Page 2
Information sources	6	Specify all databases, registers, websites, organisations, reference lists and other sources searched or consulted to identify studies. Specify the date when each source was last searched or consulted.	Page 2
Search strategy	7	Present the full search strategies for all databases, registers and websites, including any filters and limits used.	Supplementary Table S3
Selection process	8	Specify the methods used to decide whether a study met the inclusion criteria of the review, including how many reviewers screened each record and each report retrieved, whether they worked independently, and if applicable, details of automation tools used in the process.	Page 2
Data collection process	9	Specify the methods used to collect data from reports, including how many reviewers collected data from each report, whether they worked independently, any processes for obtaining or confirming data from study investigators, and if applicable, details of automation tools used in the process.	Page 2
Data items	10a	List and define all outcomes for which data were sought. Specify whether all results that were compatible with each outcome domain in each study were sought (e.g. for all measures, time points, analyses), and if not, the methods used to decide which results to collect.	Page 2
	10b	List and define all other variables for which data were sought (e.g. participant and intervention characteristics, funding sources). Describe any assumptions made about any missing or unclear information.	Page 2
Study risk of bias assessment	11	Specify the methods used to assess risk of bias in the included studies, including details of the tool(s) used, how many reviewers assessed each study and whether they worked independently, and if applicable, details of automation tools used in the process.	Pages 2-3
Effect measures	12	Specify for each outcome the effect measure(s) (e.g. risk ratio, mean difference) used in the synthesis or presentation of results.	Page 3
Synthesis methods	13a	Describe the processes used to decide which studies were eligible for each synthesis (e.g. tabulating the study intervention characteristics and comparing against the planned groups for each synthesis (item #5)).	Page 4
	13b	Describe any methods required to prepare the data for presentation or synthesis, such as handling of missing summary statistics, or data conversions.	Page 3
	13c	Describe any methods used to tabulate or visually display results of individual studies and syntheses.	Page 3
	13d	Describe any methods used to synthesize results and provide a rationale for the choice(s). If meta-analysis was performed, describe the	Page 3

Section and Topic	Item #	Checklist item	Location where item is reported
		model(s), method(s) to identify the presence and extent of statistical heterogeneity, and software package(s) used.	
	13e	Describe any methods used to explore possible causes of heterogeneity among study results (e.g. subgroup analysis, meta-regression).	Page 3
	13f	Describe any sensitivity analyses conducted to assess robustness of the synthesized results.	Page 3
Reporting bias assessment	14	Describe any methods used to assess risk of bias due to missing results in a synthesis (arising from reporting biases).	NA
Certainty assessment	15	Describe any methods used to assess certainty (or confidence) in the body of evidence for an outcome.	Page 3
**RESULTS**			
Study selection	16a	Describe the results of the search and selection process, from the number of records identified in the search to the number of studies included in the review, ideally using a flow diagram.	Figure 1<br>Page 4
	16b	Cite studies that might appear to meet the inclusion criteria, but which were excluded, and explain why they were excluded.	Page 4
Study characteristics	17	Cite each included study and present its characteristics.	Tables 1-2<br>Pages 5-11
Risk of bias in studies	18	Present assessments of risk of bias for each included study.	Supplementary Tables S4-S7<br>Page 3-4, 9
Results of individual studies	19	For all outcomes, present, for each study: (a) summary statistics for each group (where appropriate) and (b) an effect estimate and its precision (e.g. confidence/credible interval), ideally using structured tables or plots.	Figures 2-3<br>Pages 12-13
Results of syntheses	20a	For each synthesis, briefly summarise the characteristics and risk of bias among contributing studies.	Pages 9-10
	20b	Present results of all statistical syntheses conducted. If meta-analysis was done, present for each the summary estimate and its precision (e.g. confidence/credible interval) and measures of statistical heterogeneity. If comparing groups, describe the direction of the effect.	Pages 9-10
	20c	Present results of all investigations of possible causes of heterogeneity among study results.	Page 10
	20d	Present results of all sensitivity analyses conducted to assess the robustness of the synthesized results.	Page 10
Reporting biases	21	Present assessments of risk of bias due to missing results (arising from reporting biases) for each synthesis assessed.	NA
Certainty of evidence	22	Present assessments of certainty (or confidence) in the body of evidence for each outcome assessed.	Page 10
**DISCUSSION**			
Discussion	23a	Provide a general interpretation of the results in the context of other evidence.	Page 10
	23b	Discuss any limitations of the evidence included in the review.	Pages 11-12
	23c	Discuss any limitations of the review processes used.	Page 11-12
	23d	Discuss implications of the results for practice, policy, and future research.	Page 12
**OTHER INFORMATION**			

Section and Topic	Item #	Checklist item	Location where item is reported
Registration and protocol	24a	Provide registration information for the review, including register name and registration number, or state that the review was not registered.	Page 2
	24b	Indicate where the review protocol can be accessed, or state that a protocol was not prepared.	Page 2
	24c	Describe and explain any amendments to information provided at registration or in the protocol.	NA
Support	25	Describe sources of financial or non-financial support for the review, and the role of the funders or sponsors in the review.	Page 12
Competing interests	26	Declare any competing interests of review authors.	Page 12
Availability of data, code and other materials	27	Report which of the following are publicly available and where they can be found: template data collection forms; data extracted from included studies; data used for all analyses; analytic code; any other materials used in the review.	Page 12

Supplementary Table S2. MOOSE checklist

Item No	Recommendation	Reported on Page No
Reporting of background should include		
1	Problem definition	1
2	Hypothesis statement	2
3	Description of study outcome(s)	2
4	Type of exposure or intervention used	2
5	Type of study designs used	2
6	Study population	2
Reporting of search strategy should include		
7	Qualifications of searchers (eg, librarians and investigators)	2
8	Search strategy, including time period included in the synthesis and key words	2
9	Effort to include all available studies, including contact with authors	2
10	Databases and registries searched	2
11	Search software used, name and version, including special features used (eg, explosion)	3
12	Use of hand searching (eg, reference lists of obtained articles)	2
13	List of citations located and those excluded, including justification	4, Table 1
14	Method of addressing articles published in languages other than English	2
15	Method of handling abstracts and unpublished studies	2
16	Description of any contact with authors	NA
Reporting of methods should include		
17	Description of relevance or appropriateness of studies assembled for assessing the hypothesis to be tested	2
18	Rationale for the selection and coding of data (eg, sound clinical principles or convenience)	2
19	Documentation of how data were classified and coded (eg, multiple raters, blinding and interrater reliability)	2
20	Assessment of confounding (eg, comparability of cases and controls in studies where appropriate)	NA
21	Assessment of study quality, including blinding of quality assessors, stratification or regression on possible predictors of study results	3
22	Assessment of heterogeneity	3
23	Description of statistical methods (eg, complete description of fixed or random effects models, justification of whether the chosen models account for predictors of study results, dose-response models, or cumulative meta-analysis) in sufficient detail to be replicated	3
24	Provision of appropriate tables and graphics	Tables 1-2 Figures 1-3
Reporting of results should include		
25	Graphic summarizing individual study estimates and overall estimate	Figures 2-3

26	Table giving descriptive information for each study included	Tables 1-2
27	Results of sensitivity testing (eg, subgroup analysis)	10
28	Indication of statistical uncertainty of findings	9-10
Reporting of discussion should include		
29	Quantitative assessment of bias (eg, publication bias)	3
30	Justification for exclusion (eg, exclusion of non-English language citations)	12
31	Assessment of quality of included studies	10<br>Supplementary Figures S4-S7
Reporting of conclusions should include		
32	Consideration of alternative explanations for observed results	NA
33	Generalization of the conclusions (ie, appropriate for the data presented and within the domain of the literature review)	10
34	Guidelines for future research	11
35	Disclosure of funding source	12

Supplementary Table S3. Search strategy for identifying observational studies on Pubmed

**Search**	Query
**#1**	DASH diet [MeSH Terms]
**#2**	DASH diet [Title/Abstract]
**#3**	DASH [Title/Abstract]
**#4**	Dietary score [Title/Abstract]
**#5**	Dietary adherence [Title/Abstract]
**#6**	1-5/OR
**#7**	Blood pressure [Title/Abstract]
**#8**	Blood pressure [MeSH Terms]
**#9**	Hypertension [MeSH Terms]
**#10**	Hypertension [MeSH Tems]
**#11**	7-10/OR
**#12**	#6 AND #11
**The last search was performed in November 2022**	

Supplementary Table S4. Quality assessment using the Newcastle-Ottawa scale for cross-sectional studies

Study ID	Selection Bias Assessment (Maximun 5 Stars)								Comparability (Maxnimum 2 Stars)		Outcome (Maximum 3 Stars)				Total Score (Maximum 10 Stars)
	Representativeness of the Sample		Sample Size		Non-Responders		Ascertainment of the Exposure (Risk Factor)		Confounding Factors are Controlled		Assessment of the Outcome		Statistical test		
	Selection	Score	Selection	Score	Selection	Score	Selection	Score	Selection	Score	Selection	Score	Selection	Score	
Cherfan et al., 2019	a	1	a	1	b	0	a	2	a	1	b	2	a	1	8
Kanauchi M. & Kanauchi K., 2015	c	0	a	1	b	0	b	1	a	1	b	2	a	1	6
Yang et al., 2022	a	1	a	1	b	0	a	2	a	1	b	2	a	1	8

Supplementary Table S5. Quality assessment using the Newcastle-Ottawa scale for cohort studies

Study ID	Selection Bias Assessment (Maximun 4 Stars)								Comparability (Maxnimum 1 Star)		Outcome (Maximum 3 Stars)						Total Score (Maximum 10 Stars)
	Representativeness of the Exposed Cohort		Selection of the Non-Exposed Cohort		Ascertainment of Exposure		Demonstration that outcome of Interest was not Present at Start of Study		Comprability of Cohorts on the Basis of the Design or Analysis		Assessment of the Outcome		Was Follow-Up Long Enopugh for Outcomes to Occur		Adequacy of Follow-Up of Cohorts		
	Selection	Score	Selection	Score	Selection	Score	Selection	Score	Selection	Score	Selection	Score	Selection	Score	Selection	Score	
Bai et al., 2016	b	1	a	1	e	0	a	1	b	1	b	1	a	1	d	0	6
Camoes et al., 2010	b	1	a	1	b	1	a	1	b	1	b	1	a	1	d	0	7
Folsom et al., 2007	b	1	a	1	c	0	a	1	b	1	b	1	a	1	c	0	6
Francisco et al., 2020	b	1	a	1	b	1	a	1	b	1	b	1	a	1	b	1	8
Jiang et al., 2015	a	1	a	1	b	1	a	1	b	1	b	1	a	1	d	0	7
Lelong et al., 2017	b	1	a	1	c	0	a	1	b	1	b	1	a	1	d	0	6
Li et al., 2016	a	1	a	1	b	1	a	1	b	1	b	1	a	1	d	0	7
Schulze et al., 2003	d	0	a	1	c	0	a	1	b	1	b	1	a	1	d	0	5
Toledo et al., 2009	b	1	a	1	b	1	a	1	b	1	b	1	a	1	d	0	8

Supplementary Table S6. Quality assessment using the JBI checklist for cross-sectional studies

StudyID	Q1	Q2	Q3	Q4	Q5	Q6	Q7	Q8
Cherfan et al., 2019	Y	N	Y	Y	Y	Y	Y	Y
Kanauchi et al., 2015	Y	N	Y	Y	Y	Y	Y	Y
Yang et al., 2022	N	N	Y	Y	Y	Y	Y	Y
N: No; Y: Yes								

Supplementary Table S7. Quality assessment using the JBI checklist for cohort studies

StudyID	Q1	Q2	Q3	Q4	Q5	Q6	Q7	Q8	Q9	Q10	Q11
Bai et al., 2016	U	Y	Y	Y	Y	Y	Y	Y	U	U	Y
Camoes et al., 2010	U	Y	Y	Y	U	Y	Y	Y	N	N	Y
Folsom et al., 2007	U	Y	Y	Y	Y	Y	Y	U	N	U	Y
Fransisco et al., 2020	U	Y	Y	Y	Y	Y	Y	N	U	N	Y
Jiang et al., 2015	U	Y	Y	Y	Y	Y	Y	U	U	U	Y
Lelong et al., 2017	U	Y	Y	Y	Y	Y	Y	N	N	N	Y
Li et al., 2016	U	Y	Y	Y	Y	Y	Y	Y	U	U	Y
Schulze et al., 2003	U	Y	Y	Y	Y	Y	Y	U	N	N	Y
Toledo et al., 2010	U	Y	Y	Y	Y	y	y	N	U	U	Y
N: No; U: Unclear; Y: Yes											

Supplementary Figure S1. Subgroup analysis for the cohort studies based on hypertension diagnosis

Study	logHR	SE(logHR)	HR	95%-CI	Weight (common)	Weight (random)
**Hypertension Diagnosis = SBP≥140 or DBP≥90 or antihypertensive medication use**						
Bai et al., 2017	-0.1625	0.0751	0.85	[0.73; 0.98]	17.2%	16.8%
Camões et al., 2010	-0.1744	0.2115	0.84	[0.55; 1.26]	2.2%	5.2%
Francisco et al., 2020	-0.2107	0.1279	0.81	[0.63; 1.04]	5.9%	10.5%
Jiang et al., 2015	-0.0726	0.1137	0.93	[0.73; 1.14]	7.5%	11.9%
Schulze et al., 2003	-0.3567	0.2657	0.70	[0.42; 1.19]	1.4%	3.6%
**Common effect model**			**0.85**	**[0.77; 0.95]**	**34.2%**	**--**
**Random effects model**			**0.85**	**[0.77; 0.95]**	**--**	**48.0%**
Heterogeneity: $I^2 = 0\%$, $\tau^2 = 0$, $p = 0.86$						
**Hypertension Diagnosis = Self-report of hypertension**						
Folsom et al., 2007	-0.0305	0.0528	0.97	[0.87; 1.07]	34.9%	20.1%
Lelong et al., 2017	-0.4155	0.0656	0.66	[0.58; 0.75]	22.6%	18.2%
Li et al., 2016	-0.3285	0.1121	0.72	[0.58; 0.90]	7.7%	12.1%
Toledo et al., 2010	-0.7340	0.4201	0.48	[0.21; 1.09]	0.6%	1.6%
**Common effect model**			**0.82**	**[0.76; 0.88]**	**65.8%**	**--**
**Random effects model**			**0.75**	**[0.60; 0.95]**	**--**	**52.0%**
Heterogeneity: $I^2 = 88\%$, $\tau^2 = 0.0374$, $p < 0.01$						
**Common effect model**			**0.83**	**[0.78; 0.88]**	**100.0%**	**--**
**Random effects model**			**0.81**	**[0.73; 0.90]**	**--**	**100.0%**

Hazard Ratio

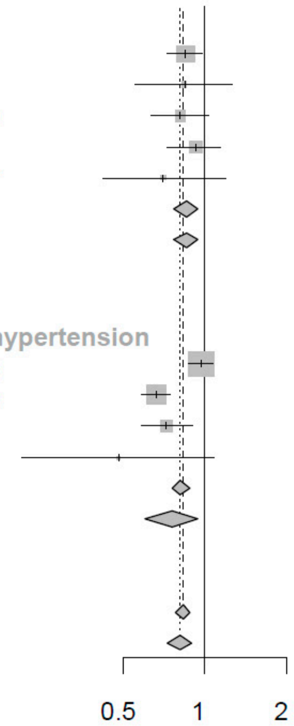

Heterogeneity: $I^2 = 69\%$, $\tau^2 = 0.0119$, $p < 0.01$
Test for subgroup differences (common effect): $\chi^2_1 = 0.45$, df = 1 ($p = 0.50$)
Test for subgroup differences (random effects): $\chi^2_1 = 0.91$, df = 1 ($p = 0.34$)

Supplementary Figure S2. Sensitivity analysis by removing cohort studies with a NOS score <7

Study	logHR	SE(logHR)	Hazard Ratio	HR	95%-CI	Weight (common)	Weight (random)
Camões et al., 2010	-0.1744	0.2115		0.84	[0.55; 1.26]	9.1%	9.4%
Francisco et al., 2020	-0.2107	0.1279		0.81	[0.63; 1.04]	24.8%	25.0%
Jiang et al., 2015	-0.0726	0.1137		0.93	[0.73; 1.14]	31.4%	31.2%
Li et al., 2016	-0.3285	0.1121		0.72	[0.58; 0.90]	32.3%	32.0%
Toledo et al., 2010	-0.7340	0.4201		0.48	[0.21; 1.09]	2.3%	2.4%
**Common effect model**				**0.81**	**[0.71; 0.91]**	**100.0%**	**--**
**Random effects model**				**0.81**	**[0.71; 0.92]**	**--**	**100.0%**
**Prediction interval**					**[0.64; 1.01]**		

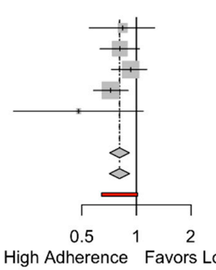

Heterogeneity: $I^2 = 4\%$, $\tau^2 = 0.0008$, $p = 0.39$
